# Temporal Trends and Disparities in Suicidal Behaviors by Sex and Sexual Identity Among Asian American Adolescents

**DOI:** 10.1001/jamanetworkopen.2021.4498

**Published:** 2021-04-16

**Authors:** Yunyu Xiao, Wenhua Lu

**Affiliations:** 1 School of Social Work, Indiana University–Purdue University Indianapolis, Indianapolis; 2 School of Social Work, Indiana University Bloomington, Bloomington; 3 Department of Community Health and Social Medicine, School of Medicine, City University of New York, New York

## Abstract

**Importance:**

Although suicide is the second leading cause of death among Asian American adolescents, there is a dearth of studies examining overall and possible sex and sexual orientation disparities in the trends in suicidal behaviors among Asian American adolescents. Such information is crucial to inform targeted efforts of suicide prevention among Asian American adolescents.

**Objective:**

To examine temporal trends and sex and sexual orientation disparities in trends of nonfatal suicidal behaviors in Asian American adolescents from 1991 through 2019.

**Design, Setting, and Participants:**

This cross-sectional study used data from the national Youth Risk Behavior Survey from 1991 through 2019, analyzing a representative sample of US adolescents in grades 9 through 12 using a 3-stage cluster-sampling design. Data were analyzed from October through November 2020.

**Exposures:**

Calendar year, sex, sexual identity, sex of sexual contact, and interaction terms of these factors.

**Main Outcomes and Measures:**

Crude prevalence and annual percentage changes (APCs) in self-reported suicidal ideation, suicide plan, suicide attempts, and injury by suicide attempt for the overall sample and by sex, sexual identity, and sex of sexual contacts were calculated. Sexual minorities were defined as individuals whose sexual identity was gay or lesbian, bisexual, or not sure.

**Results:**

Among 7619 Asian Americans who participated in the Youth Risk Behavior Survey from 1991 to 2019 (mean [SD] age, 16.09 [1.29] years; 3760 [47.1%] female adolescents), 1576 individuals completed the sexual identity and behaviors questions after 2015 (mean [SD] age, 15.97 [1.28] years; 810 [49.2%] female adolescents). From 2009 through 2019, there was a 1.3-fold (95% CI, −0.8 to 3.3; *P* = .22) increase in suicide attempts and a 1.7-fold (95% CI, −2.6 to 5.9; *P* = .45) increase in injury by suicide attempt among Asian American female adolescents, although these increases were not statistically significant. Among 39 Asian American adolescents who identified as gay, lesbian, or bisexual or who were attracted to and had sexual contact with partners of the same sex or both sexes, compared with 1556 Asian American adolescents who were heterosexual, prevalence was greater for suicidal ideation (24 individuals [68.2%] vs 223 individuals [15.0%]; *P* < .001), suicide plan (15 individuals [57.7%] vs 179 individuals [11.9%]; *P* < .001), suicide attempts (14 individuals [41.0%] vs 74 individuals [5.5%]; *P* < .001), and injury by suicide attempt (5 individuals [17.6%] vs 23 individuals [1.7%]; *P* < .001) between 2015 and 2019. These sexual minorities identified by sexual identity and sexual contact also had an increasing rate over this period in suicide plan (APC, 10.5%; 95% CI, 4.4% to 16.9%; *P* < .001).

**Conclusions and Relevance:**

This study found significant increases in rates of suicide plan among Asian American adolescents who were sexual minorities identified by sexual identity and sexual contact together. These findings suggest that suicide screening needs to inquire about sexual minority status in terms of sexual identity and sex of sexual contact when identifying Asian American adolescents who are at risk for suicidal behaviors. Culturally relevant suicide-prevention programs addressing unique risk and protective factors, racial discrimination, and sexual orientation–related stigma may be needed for Asian American adolescents.

## Key Points

**Question:**

What are the trends and within-group disparities in self-reported suicidal behaviors among Asian American adolescents from 1991 through 2019?

**Findings:**

In this cross-sectional study with a nationally representative sample of 7619 Asian American adolescents, increases in the prevalence of suicide attempts and injury by suicide attempt were observed among female adolescents from 2009 to 2019, although these increases were not statistically significant. A statistically significant increase in suicide plan was found among Asian American adolescents with partners of the same sex or both sexes.

**Meaning:**

These findings suggest that suicide disparities among Asian American adolescents warrant attention, and that culturally tailored suicide prevention that reduces racial discrimination and sexual orientation–related stigma may help prevent suicide in this group.

## Teaser

This cross-sectional study uses data from the national Youth Risk Behavior Survey to investigate the trends and within-group disparities in 4 suicidal behaviors by sex and sexual identity among Asian American adolescents from 1991 through 2019.

## Introduction

Suicide is the second leading cause of death among Asian American adolescents in the US.^1^ In 2018, approximately 8% of Asian American adolescents aged 12 to 18 years died by suicide.^1^ Studies from 2019^2,3,4^ found between-group racial/ethnic disparities in suicidal behaviors, with a narrowing gap between Black and White populations and male and female populations. Less is known, however, about the trends in suicidal behaviors among Asian American adolescents over time.^5^ To improve precision in suicide prevention in the populations with the greatest risk, it is critical to monitor trends and within-group disparities in suicidal behaviors among Asian Americans adolescents.^6,7^

Adolescents who are sexual minorities (defined as individuals who self-identify as gay, lesbian, or bisexual or who are attracted to or have sexual contact with people of the same sex) bear a disproportionate risk of suicidal behaviors compared with heterosexual individuals.^8,9^ Results from 2 studies from 2020^9,10^ highlight the need to recognize the diversity among youth who are sexual minorities, given that a single-dimension definition may obscure within-group disparities and accurate estimation of suicide risks.^10,11^ Owing to the strong cultural stigma toward sexual minorities, Asian American adolescents who are sexual minorities may be less likely to disclose their nonheterosexual identities, same-sex or both-sex attraction, or sexual behaviors.^12^ Co-occurrence of racial discrimination and sexual-orientation stigma could be associated with increased suicide risks and health disparities in suicide.^13^

Thus far, trends of suicidal behaviors in Asian American youth who are sexual minorities have not been reported, to our knowledge. It is unknown whether living at the intersections of multiple marginalized identities, including sex, sexual identity, and sexual behavior contacts among racial minorities, would further reinforce culturally unique minority stress^14^ and reflect the epidemiological outcomes of disparities in suicidal behaviors among Asian American adolescents. Furthermore, most existing literature has relied on convenience samples, partially owing to the unavailability of nationally representative population-based data.^15,16^

To our knowledge, this study presents the first nationally representative analysis of temporal trends in suicidal behaviors among Asian American adolescents over 28 years, from 1991 through 2019. With the benefit of added sexual minority identification questions in the National Youth Risk Behavior Survey (YRBS) in 2015 and recently released 2019 data, a secondary objective was to identify sex and sexual minority disparities in suicidal trends among Asian American adolescents to identify high-risk subgroups to be prioritized in interventions.

## Methods

This cross-sectional study was determined by Indiana University to be exempt from institutional review board review because it used publicly available data.^17^ Written informed consent for participation in the YRBS was obtained from the parents or legal guardians of adolescents. We followed the Strengthening the Reporting of Observational Studies in Epidemiology (STROBE) reporting guideline for cross-sectional studies.

### Data and Participants

The Youth Risk Behavior Surveillance System (YRBSS) was established by the Centers for Disease Control and Prevention to measure health risk behaviors among US adolescents.^18^ Data for this system were drawn from the combined data sets of the national survey (ie, YRBS), including 217 340 unweighted surveys conducted from 1991 through 2019 nationwide. Using a 3-stage cluster-sampling design, the YRBS produced biannual nationally representative samples of high school students in grades 9 through 12 among public and private schools in the 50 states and the District of Columbia. Students self-administered computer-scannable questionnaires anonymously and voluntarily. More details on study design and sample recruitment are reported elsewhere.^19^

In this cross-sectional study, overall trends in suicidal behaviors were analyzed among 7619 adolescents who self-identified as Asian Americans in the YRBS 1991 through 2019 (ie, sample 1). Trends in within-group changes were estimated in the subsample of 1576 Asian American adolescents who provided information about their sex and sexual orientation from 2015 through 2019 (ie, sample 2); 2015 was the first year when data were collected on sexual identity and sex of sexual contacts. The overall response rates ranged from 60.3% (13 677 of 17 025 students responding [80.3%] × 136 of 181 schools responding [75.1%]) to 71.4% (16 460 of 18 573 students responding [88.6%] × 158 of 196 schools responding [80.6%]) across the survey years for variables included in this study. Participant demographic characteristics are available in eTable 1 in the Supplement.

### Measures

Outcomes included 4 self-reported suicidal behaviors measured by responses to associated questions: (1) suicide ideation (“During the past 12 months, did you ever seriously consider attempting suicide?”), (2) suicide plan (“During the past 12 months, did you make a plan about how you would attempt suicide?”), (3) suicide attempts (“During the past 12 months, how many times did you actually attempt suicide?”), and (4) injury by suicide attempt (“If you attempted suicide during the past 12 months, did any attempt result in an injury, poisoning, or overdose that had to be treated by a doctor or nurse?”). Response options were dichotomized into *yes* or *no* for analysis. Suicidality items were previously found to be reliable and valid.^20,21^

The main exposure was time in years. Linear and quadratic terms between study periods were examined. Sex groups were self-reported biological sex (ie, male or female). Sexual orientation was assessed using 2 measures, with associated questions: self-reported sexual identity (“Which of the following best describes you?”) and sex of sexual contacts (“During your life, with whom have you had sexual contact?”). Responses were dichotomized into *heterosexual* and *sexual minority* (ie, gay or lesbian, bisexual, or not sure) for sexual identity and *opposite-sex sexual contacts* and *any same-sex or both-sexes sexual contacts* for sexual contacts. Student grade levels were included as controlled variables.

### Statistical Analysis

We conducted 3 main analyses. First, we reported and visualized the trends of crude rates of suicidal behaviors among all Asian Americans from 1991 through 2019. Second, we derived annual percentage changes (APC) and average annual percentage changes (AAPC) using joinpoint regression^22^ to identify the years at which significant changes in rates of suicide behaviors occurred over the study period and the size of these changes. Trends for each suicidal behavior were stratified by sex, sexual orientation, and intersections between sex and sexual orientation. Significant changes in the slope of trends were presented using linear segments (ie, joinpoints). Nonsignificant changes in trends were fitted as straight lines. Statistical differences in slope between sex, sexual orientation, and intersectionality groups were tested. Third, we estimated adjusted odds ratios (ORs) using logistic regression models in which the survey year was treated as a continuous variable, adjusting for grade levels. Inclusion of the survey year variable in the regression models allowed us to estimate the changes in suicidal behaviors in a given year relative to the previous few years. Linear and nonlinear (ie, adding quadratic terms of year) trends were evaluated. Intersectional terms of sex, sexual identity, and sex of sexual contacts were constructed (ie, sex *×* sexual identity, sex *×* sex of sexual contacts, and sexual identity *×* sex of sexual contacts) to account for within-group differences in suicide trends (ie, differences in the rate of change).^2,23^

Statistical significance was determined by a 2-sided *P* value < .05 with Wald χ^2^ tests using design-adjusted coefficient variance-covariance matrices. All analyses incorporated complex sampling design and survey weight to obtain US nationally representative estimates, accounting for probabilities of sample selection, survey nonresponse, and oversampling of Black and Hispanic students. Analyses and visualization were conducted using Stata statistical software version 16 (StataCorp), Joinpoint statistical software version 4.8.0.1 (National Cancer Institute),^22^ and Python programming language version 3.9 (Python Software Foundation). Data were analyzed from October through November 2020.

## Results

### Overall Trends in Suicidal Behaviors 1991-2019

**Figure 1.**
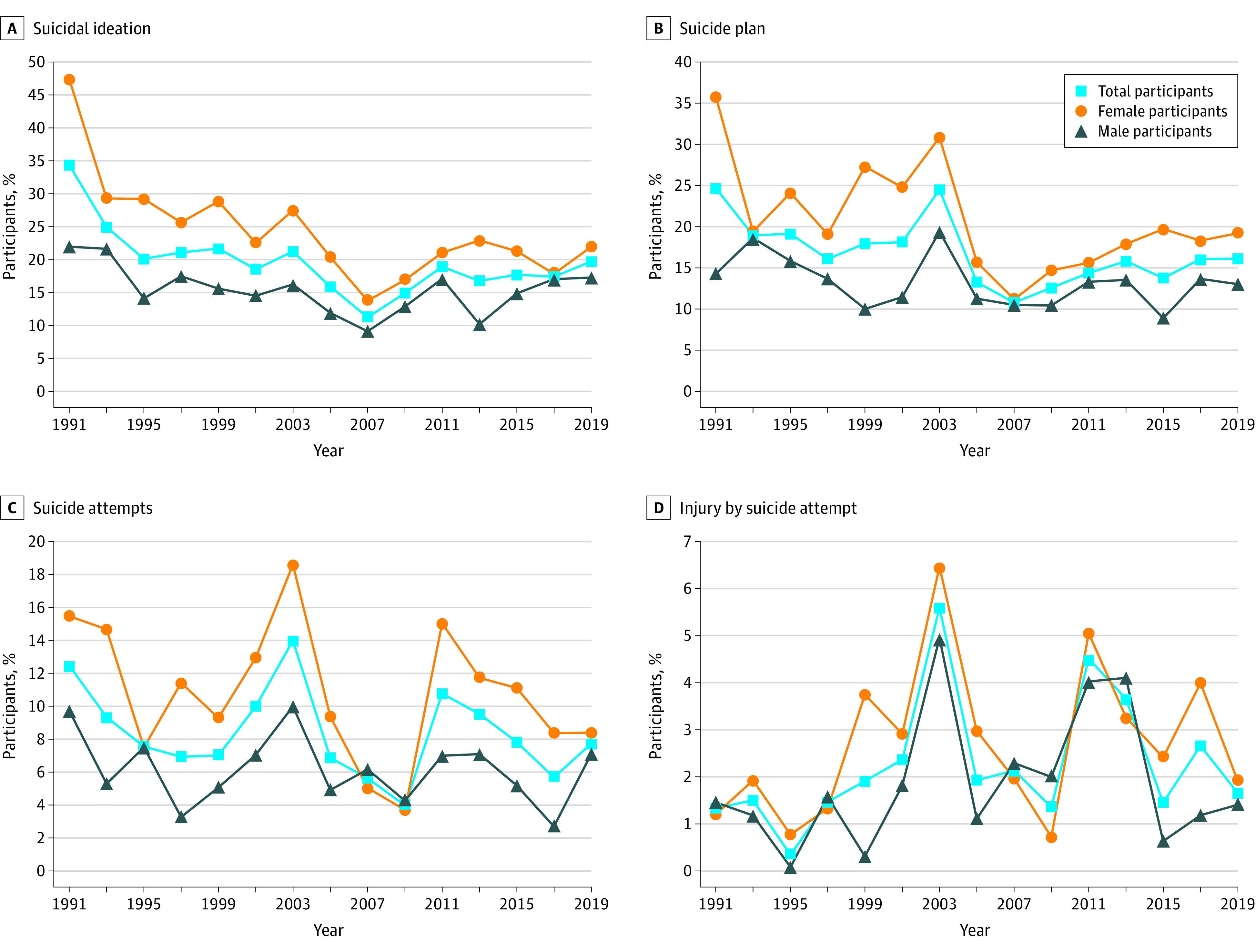
Trends in Rates of Suicidal Behaviors

Among 7619 Asian Americans who participated in the YRBS 1991-2019 (ie sample 1; mean [SD] age, 16.09 [1.29] years; 3760 [47.1%] female adolescents), 12-month prevalence rates ranged from 53 of 418 adolescents (11.2%) in 2007 to 140 of 378 adolescents (34.4%) in 1991 for suicidal ideation, from 48 of 418 adolescents (10.8%) in 2007 to 93 of 368 adolescents (24.7%) in 1991 for suicide plan, from 36 of 681 adolescents (4.0%) in 2009 to 38 of 349 adolescents (14.0%) in 2003 for suicide attempts, and from 21 of 241 adolescents (0.3%) in 1995 to 38 of 349 adolescents (5.6%) in 2003 for injury by suicide attempt (Figure 1; eTable 1 and 2 in the Supplement). The peak prevalence rates for suicide attempts and injury by suicide attempt among Asian American adolescents were observed in 2003, followed by a linear decrease from 2003 to 2009 and then a second peak in 2011. The increase in prevalence rates of all suicidal behaviors among Asian American adolescents increased in the recent decade (ie, 2009-2019), ranging from 22.4% for injury by suicide attempt to 94.4% (for suicide attempts).

Overall, there was a significant decreasing trend in prevalence of suicide plan (AAPC, −1.5%; 95% CI, −2.8% to −0.2%; *P* < .001); there were also decreasing trends in suicidal ideation (AAPC, −2.4%; 95% CI, −5.1% to 0.3%; *P* = .10) and suicide attempts (AAPC, −1.0%; 95% CI, −3.0% to 1.0%; *P* = .30), but these changes were not statistically significant. The rate of injury by suicide attempt increased by 1.5% (95% CI, −3.0% to 6.3%; *P* < .50) per year over the 1991 through 2019 period, but this change was not statistically significant (eFigure 1 in the Supplement).

### Sex Disparities

Among 1576 individuals who completed the sexual identity and behaviors questions after 2015 (ie, sample 2), mean (SD) age was 15.97 (1.28) years and 810 (49.2%) individuals were female. Increases in prevalence were observed among female and male Asian American adolescents from 2009 through 2019 for suicidal ideation, suicide plan, and injury by suicide attempt, although these increases were not statistically significant (Figure 1). The prevalence of injury by suicide attempt among female Asian American adolescents increased from 4 of 367 individuals (0.7%) in 2009 to 4 of 206 individuals (1.9%) in 2019, for a 1.7-fold increase (95% CI, −2.6 to 5.9; *P* = .45) (eTable 2 in the Supplement), whereas the prevalence decreased among male counterparts, from 6 of 314 individuals (2.0%) in 2009 to 1 of 206 individuals (1.4%) in 2019, for a 0.3-fold decrease (95% CI, −1.83 to 1.24; *P* = .70). Neither of these changes were statistically significant. Similarly, suicide attempts among female Asian American adolescents increased from 20 of 367 individuals (3.7%) in 2009 to 16 of 235 individuals in 2019 (8.4%), for a 1.3-fold change (95% CI, −0.8 to 3.3; *P* = .22), compared with a change from 16 of 314 individuals (4.3%) in 2009 to 19 of 240 individuals (7.1%) in 2019, for an increase of 0.67-fold among male adolescents (95% CI, −0.92 to 2.26; *P* = .41). Neither of these changes were statistically significant.

The prevalence of suicide attempts increased from 30 of 193 female adolescents (15.5%) in 1991 to 21 of 162 female adolescents (18.6%) in 2003, for an increase of 20.3%, and from 14 of 181 male adolescents (9.7%) in 1991 to 17 of 184 male adolescents (10.0%) in 2003, for an increase of 2.6%. The prevalence of injury by suicide attempt increased from 5 of 193 female adolescents (1.2%) in 1991 to 8 of 159 female adolescents (6.5%) in 2003, for an increase of 436.8%, and from 1 of 181 male adolescents (1.5%) in 1991 to 7 of 182 male adolescents (5.0%) in 2003, for an increase of 240.1%.

**Figure 2.**
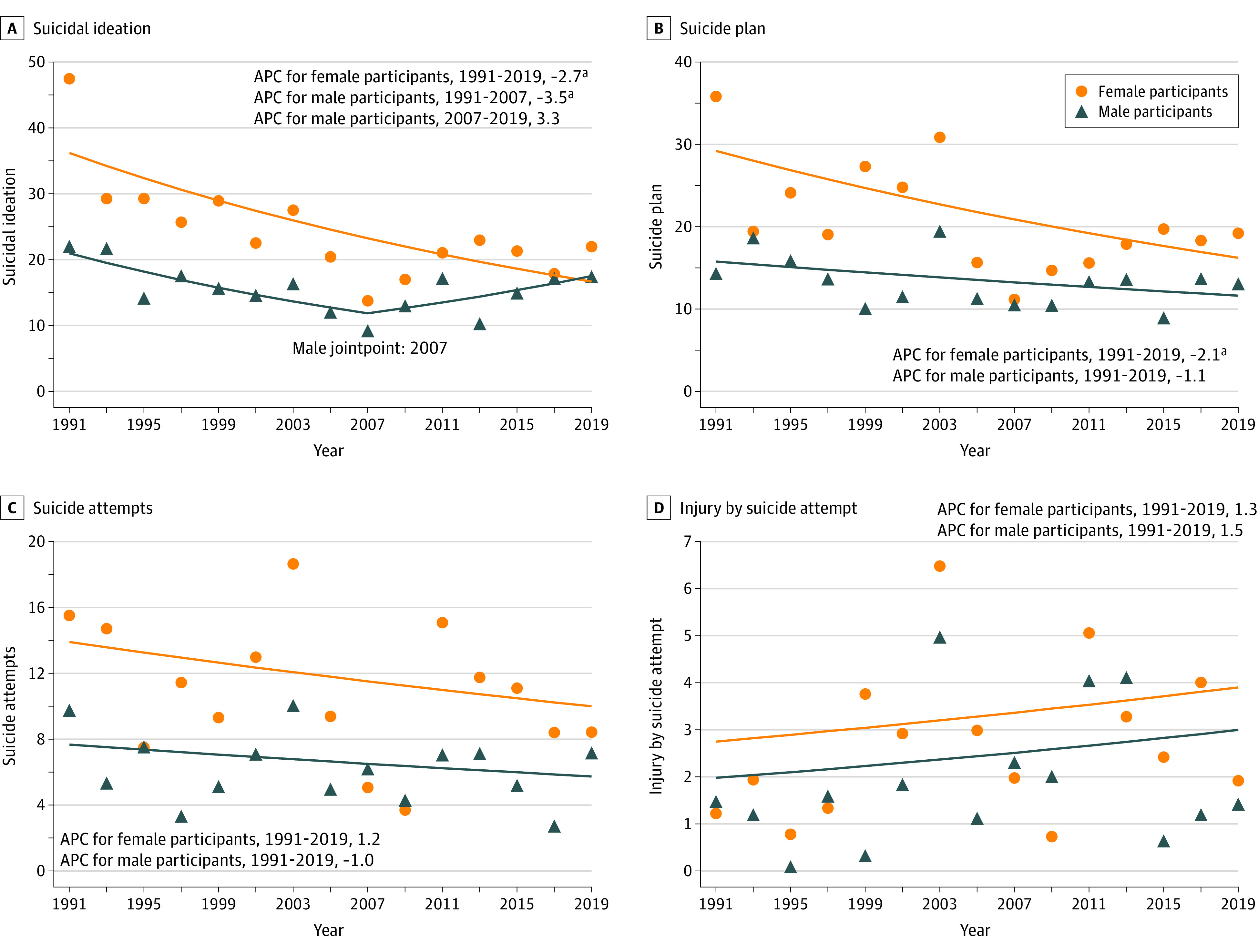
Annual Percentage Change (APC) in Suicidal Behaviors by Sex ^a^*P* < .001.

**Figure 3.**
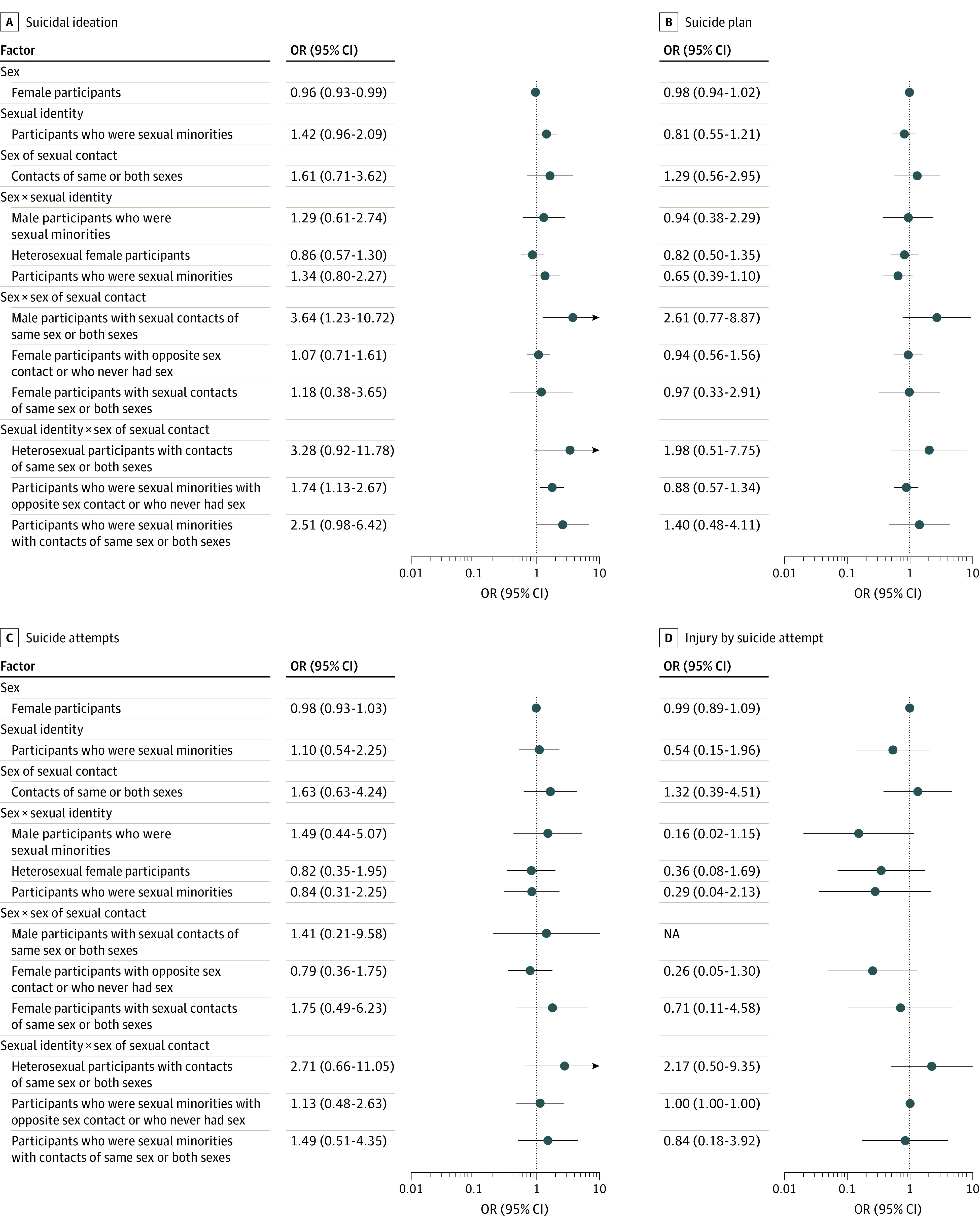
Logistic Regressions of Trends in Suicidal Behaviors by Sex, Sexual Identity, and Intersectionality NA indicates not applicable.

In joinpoint regression, we found that the rate of suicidal ideation among female adolescents decreased significantly from 1991 to 2019 (APC, −2.7%; 95% CI, −4.1% to −1.4%; *P* < .001). Among male adolescents, suicidal ideation decreased significantly from 1991 to 2007 (APC, −3.5%; 95% CI, −5.9% to −1.1%; *P* < .001), followed by a nonsignificant increase until 2019 (APC, 3.3%; 95% CI, −1.3% to 8.1%; *P* = .10). The rate of suicide plan decreased among both sexes, but the decrease was not statistically significant in male adolescents (female adolescents: APC, −2.1%; 95% CI, −3.7% to −0.4%; *P* < .001; male adolescents: −1.1%; 95% CI, −2.4% to 0.3%; *P* = .10). However, there was a steeper increase in injury by suicide attempt among male adolescents compared with female adolescents, although neither of the changes were statistically significant (APC, 1.5%; 95% CI, −3.8% to 7.0%; *P* = .60 vs. APC, 1.3%; 95% CI, −3.2% to 5.9%; *P* = .60) (Figure 2). In results from logistic regressions, there were higher odds of suicidal ideation among female adolescents than male adolescents (adjusted OR, 2.49; 95% CI, 1.90 to 3.26; *P* < .001), while the increasing rate has decreased over time (adjusted OR, 0.96; 95% CI, 0.93 to 0.99; *P* = .01) (Figure 3; eTable 3 in the Supplement).

### Sexual Identity Disparities

**Figure 4.**
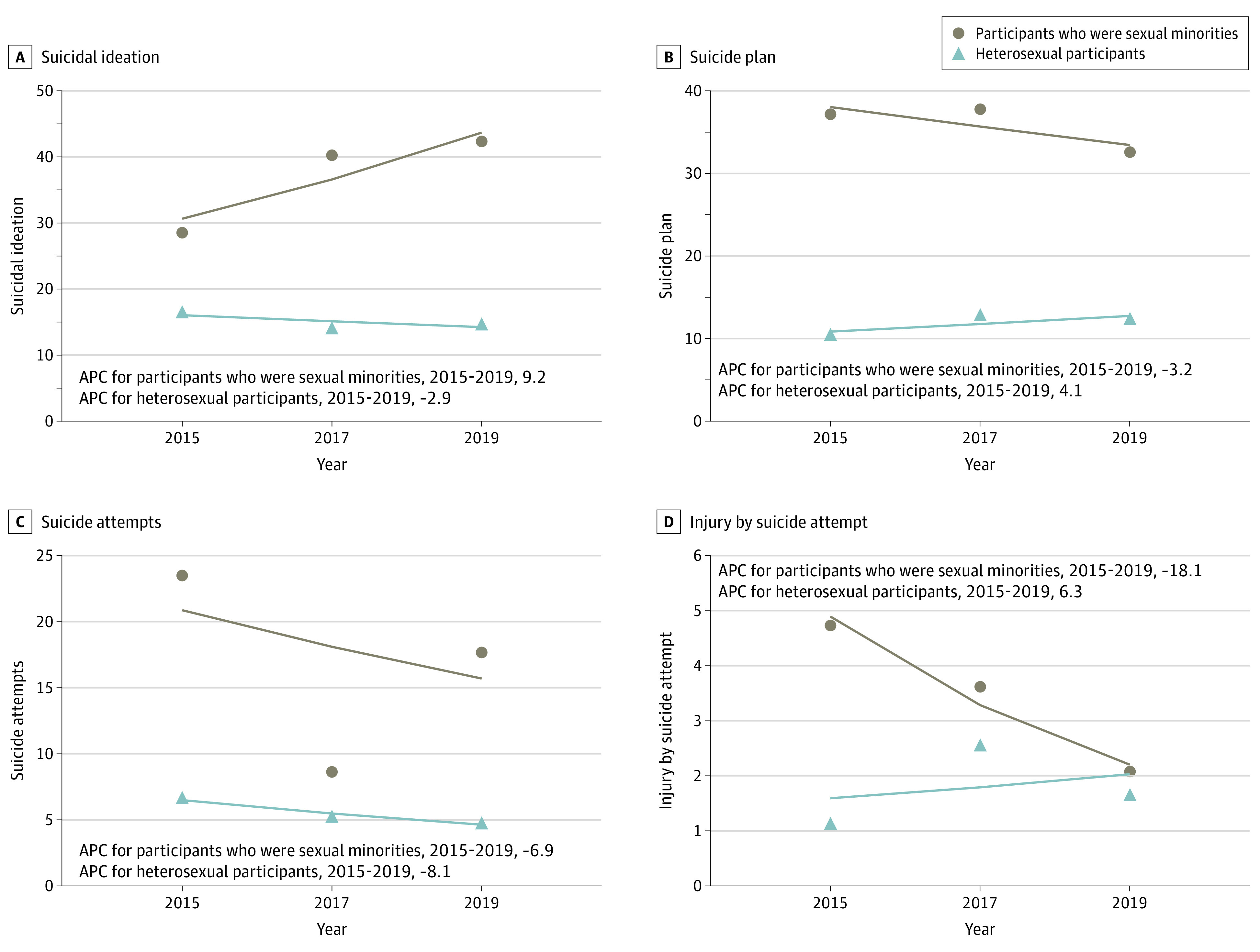
Annual Percentage Change (APC) in Suicidal Behaviors by Sexual Identity

**Figure 5.**
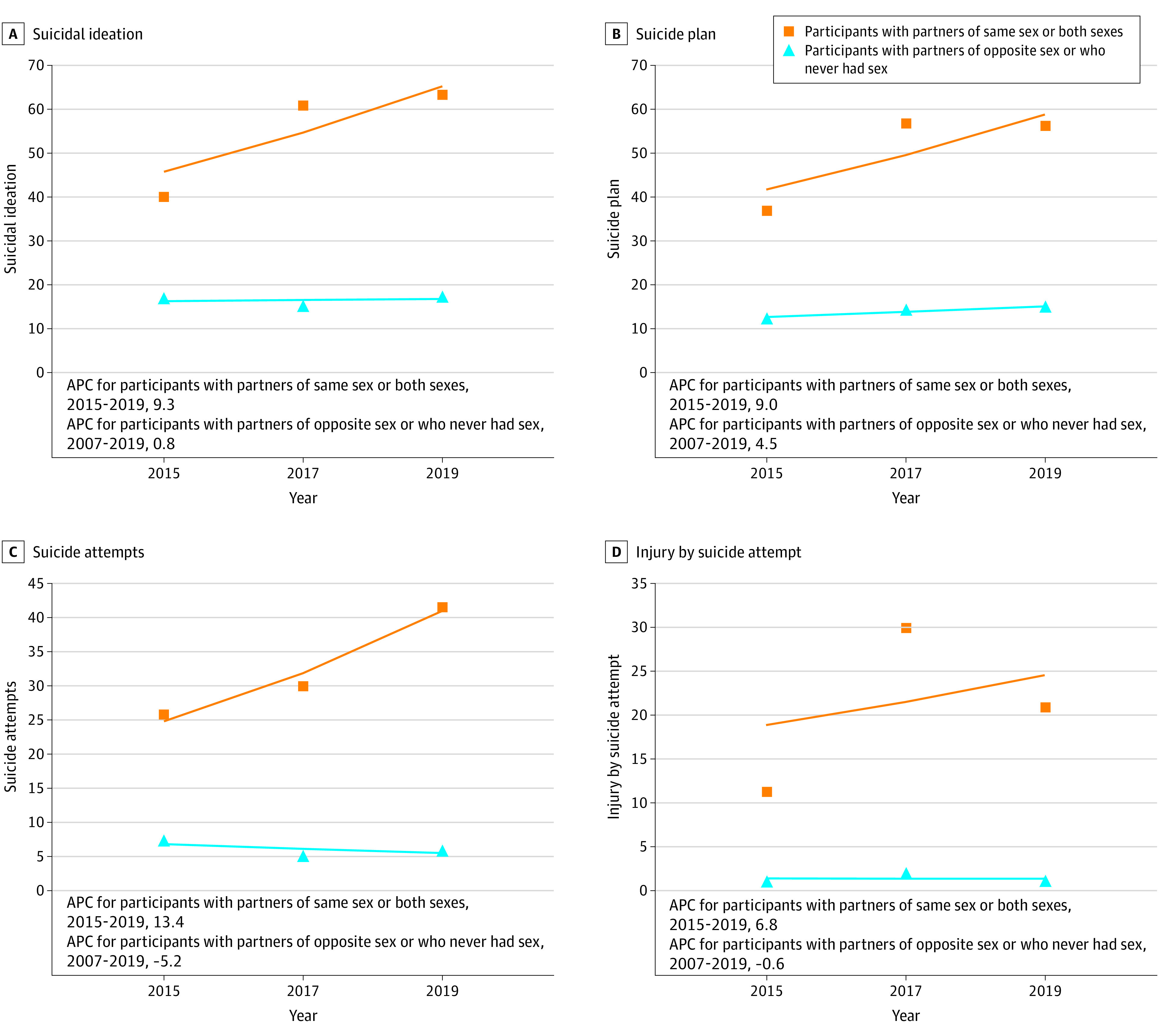
Annual Percentage Change (APC) in Suicidal Behaviors by Sex of Sexual Contacts

Prevalence rates from 2015 through 2019 for all indicators of suicidal behaviors were higher among 245 Asian American adolescents who were sexual minorities compared with 1556 Asian American adolescents who were heterosexual: suicidal ideation (90 individuals [38.7%] vs 223 individuals [15.0%]; *P* < .001), suicide attempts (34 individuals [16.3%] vs 74 individuals [5.5%]; *P* < .001), and suicide plan (75 individuals [35.0%] vs 179 individuals [11.9%]; *P* < .001). Differences between these groups for injury by suicide attempt were not statistically significant (6 individuals [3.2%] vs 23 individuals [1.7%]; *P* = .24). When measured by self-reported sexual identities (Figure 4), Asian American adolescents who were sexual minorities showed an increasing yet nonsignificant trend in suicidal ideation (APC, 9.2%; 95% CI; −37.1% to 89.6%; *P* = .30). Decreasing, yet nonsignificant, trends in other suicidal behaviors were observed regardless of sexual identity. When measured by sex of sexual contacts, Asian American adolescents who had sexual contacts of the same sex or both sexes had a 13.4% annual increase in suicide attempts (95% CI; −14.8% to 50.9%; *P* = .10) (Figure 5). In contrast, there was a decreasing trend in suicide attempts among adolescents who never had sexual contact or had sexual contact with the opposite sex (APC, −5.2%; 95% CI; −56.2% to 105.3%; *P* = .50).

### Intersectionality Across Sex and Sexual Minority Status

In the investigation of the intersection across sex and sexual identity among 1798 indivduals who reported sex and sexual identity, we found increased prevalence (SE) of suicide attempts in 2019 compared with 2015 among 77 Asian American male adolescents who identified themselves as sexual minorities (4.3%) (18.1% [36.8%] vs 15.6% [37.9%]), while suicide attempt rates (SE) were lower in 2019 vs 2015 among 168 female adolescents who were sexual minorities (9.3%) (17.52% [34.29%] vs 27.35% [49.11%]) and 746 heterosexual female adolescents (41.5%) (5.38% [20.24%] vs 9.00% [29.66%]). We also found increased prevalence of injury by suicide attempt over this period among 807 Asian American heterosexual male adolescents (44.9%) (1.7% [12.1%] vs 0.2% [4.7%]). Meanwhile, increasing trends were found in suicide plan (APC, 8.8%; 95% CI, 0.3%-18.1%; *P* < .001) and injury by suicide attempt (APC, 62.0%; 95% CI, 30.4%-101.3%; *P* < .001) among heterosexual male adolescents (eFigure 2 in the Supplement). In the investigation by sex and sex of sexual contact, we found that Asian American male adolescents who had no sex or who had sex with opposite-sex partners were 3.6-fold (95% CI, 1.23-10.72; *P* = .02) more likely to report suicidal ideation than their female counterparts over time (Figure 3; eTable 3 and eFigure 3 in the Supplement).

Among 39 Asian American adolescents who identified as gay, lesbian, or bisexual or who were attracted to and had sexual contact with partners of the same sex or both sexes, significantly greater rates were reported in all suicidal behaviors, compared with heterosexual Asian American adolescents: suicidal ideation (24 individuals [68.2%]; *P* < .001), suicide attempts (14 individuals [41.0%]; *P* < .001), suicide plan (15 individuals [57.7%]; *P* < .001), and injury by suicide attempt (5 individuals [17.6%]; *P* < .001). These sexual minorities identified by sexual identity and sexual contact also had a significantly increasing trend in suicide plan (APC, 10.5%; 95% CI; 4.4% to 16.9%; *P* < .001) and nonsignficantly increasing trends in suicidal ideation (APC, 11.3%; 95% CI; −3.6% to 28.6%; *P* = .10) and suicide attempts (APC, 8.1%; 95% CI; −87.2% to 811.4%; *P* = .70) (eFigure 4 in the Supplement). Sexual minorities who had opposite-sex or no sexual experiences also reported nonsignficantly increased suicidal ideation (APC, 16.3%; 95% CI, −40.1% to 125.8%; *P* = .20). Compared with heterosexual adolescents who never had sex or those who had opposite-sex partners, youth who were sexual minorities reported greater and increasing suicidal ideation (adjusted OR, 1.74; 95% CI, 1.13-2.67; *P* < .001) over time (Figure 3; eTable 3 in the Supplement).

## Discussion

This cross-sectional study among Asian-American adolescents found that the prevalence of suicide attempts and injury by suicide attempt increased over the study period among female adolescents, though this change was not significant, and the prevalence of suicide plan increased significantly among adolescents with sexual partners of the same sex or both sexes. We also found a significantly increased prevalence in suicidal ideation among Asian American male adolescents who had sex with same-sex partners or partners of both sexes, as well as among those who were sexual minorities who never had sex or had sexual contact with opposite-sex partners, compared with their heterosexual counterparts and those who had sex with partners of the opposite sex. To our knowledge, this cross-sectional study is the first study documenting temporal trends and sex and sexual orientation disparities in suicidal behaviors among Asian American adolescents using a nationally representative and most up-to-date data set in the US. Our results identified 2003 as a peak year of suicidal behaviors among Asian American adolescents, despite the overall decreasing trend over time. We observed an increase in the trend of suicide attempts and injury by suicide attempt but not in other suicidal behaviors among adolescents who were sexual minorities who had sex with same-sex partners or partners of both sexes. These findings suggest the need to carefully screen for Asian American adolescents at risk of suicidal behaviors.

Suicide attempts and injury by suicide attempt among Asian American adolescents increased to their highest points over the study period in 2003, with a greater increase in the crude rates among female adolescents (suicide attempts: 20.3%; injury by suicide attempt: 436.8%) than that among male adolescents (suicide attempts: 2.6%; injury by suicide attempt: 240.1%). The general trend and peak year are consistent with previous research comparing between-group trends in suicidal behaviors across racial/ethnic groups.^2^ Since 2001, several government initiatives have been issued, such as the National Suicide Prevention Lifeline, established in 2011, and the *National Strategy for Suicide Prevention*.^6,24,25^ These strategic, federal, and program initiatives were found to be associated with short-term decreases in suicide rates in the counties that employed the program’s funds for suicide prevention through public and school education.^26^ Continued efforts to reduce suicide at the structural level are needed.^13^

We found increases in suicide attempts and injury by suicide attempt among Asian American female adolescents in the recent decade, although the trends of these increases were not statistically significant. These recent increases may be associated with the rising rates of cyberbullying through social media and online gaming that started to be prevalent between 2005 and 2006.^27^ The black-box warning of antidepressants announced by the Food and Drug Administration was also found to be associated with increased suicidal behaviors through the creation of barriers for help-seeking and increases in untreated mental illness, particularly among adolescents of racial minorities.^28,29^

Distinct patterns of change in suicidal behaviors for specific groups of sex and sexual orientation across sex underscore the need to consider within-group differences in Asian American adolescent populations. Aggregating these populations into 1 group without articulating the potential differences across sex, sexual identity, and sexual contacts could mask nuanced, yet important disparities that need to be considered for tailored interventions to address the cultural stigma and minority stress faced by Asian American youth who are sexual minorities.^11,13^

Despite some decreasing trends, we found that female adolescents and adolescents who were sexual minorities consistently showed greater engagement in suicidal behaviors than their male, heterosexual counterparts and those who had opposite-sex partners. Such trends are consistent with recent observations among youth of all racial/ethnic groups who are sexual minorities.^9,10,30^ Berlan et al^31^ found that youth from both sexes who were sexual minorities were more likely to experience bullying than their heterosexual counterparts. In particular, youth who are sexual minorities often differ from their peers in physical appearance and in being gender nonconforming, which has been found to be associated with increased risks of social isolation, harassment, bullying, and other mistreatment by peers.^32,33,34^ For Asian American youth who are sexual minorities, culture-related sexual stigma and discrimination, fear of family rejection, parent-adolescent conflict, and low acculturation could further increase their minority stress and heighten risks for suicidal behaviors.^35,36,37^

Examining sexual minority status by sexual identity and behaviors underscores that relying on self-reported sexual orientation alone could mask elevated risks for suicide among Asian American adolescents. In particular, with the fear of family conflicts and cultural stigma attached to suicidal behaviors, Asian American youth who are sexual minorities may be even less likely to disclose their sexual identities while still engaging in sexual behaviors with members of the same sex or both sexes.^9^ It is possible that Asian American adolescents with suicidal thoughts who were sexual minorities and who had disclosed their sexual identities experienced a longer period of discrimination, harassment, and stigma.^38^ Accumulation of stigma and discrimination experiences has been found to be associated with increased rates of suicidal behaviors among youth who are sexual minorities.^37^ Our findings, therefore, underscore the importance of improving culturally specific screening tools to identify the intersectional identities of sex and sexual orientation among Asian American adolescents who are at increased risk of suicidal behaviors. The association of intersectionality with increased risk of suicide highlights the need for examining co-occurring latent risk profiles,^39,40^ including trauma, shame of disclosure, low sense of belongingness, experience of bullying, and low parental attachment.^37^

Findings from this study hold important public health implications. The prevalence of suicidal behaviors among Asian American adolescents remains high in recent years, with increasing rates of suicide attempts and injury by suicide attempt among female adolescents. School psychologists and social workers should be encouraged to facilitate connectedness between Asian American youth and their parents to improve prevention and early recognition of adolescent suicidal behaviors by parents. Digital suicide prevention tools, such as smartphone apps,^41^ online and mobile text message interventions (eg, caring letters suicide prevention intervention),^42^ and wearable sensors for real-time monitoring of behavioral and contextual signals may also be useful for screening risks among Asian American adolescents who are sexual minorities by reducing the barriers and reluctance associated with seeking help.^43,44,45^ More gender-sensitive language in school policies is encouraged to build a more inclusive school environment. Antibullying policies could also reduce the exposure to mistreatment and improve school climate to reduce suicide risks.^45^

### Limitations

This study has several limitations. First, due to the cross-sectional nature of YRBS, we could not determine causality in the trends. Second, YRBSS surveyed only individuals who attended schools, and thus, the data are not representative of all adolescents in the age group. Third, the measurement of suicidal behaviors and demographic characteristics are self-reported, and there is a potential for reporting bias despite the good test-retest reliability^46^ and wide use in previous studies.^39^ Fourth, there could be generational differences in the trends in suicidal behaviors, given that immigration status could be associated with different levels of acculturation in the US.^47^ However, we did not have information on the generation of the immigrants (eg, first generation) or country of origin in the current data. Future studies should examine immigration status and other confounders, including mental illness, health risk behaviors, and social networks,^39,40^ to estimate changes in suicidal behaviors among Asian American adolescents. Fifth, we focused on suicidal behaviors among adolescents self-identified as Asian Americans, without further considering within-group differences (eg, differences among Chinese Americans and Korean Americans) given that these differences were not measured in the data. Future studies should address such differences with more active data collection. Despite these limitations, to our knowledge, our study represents the first population-based investigation of sex and sexual orientation disparities in various suicidal behaviors among Asian American adolescents over time.

## Conclusions

This cross-sectional study found a greater increase in crude prevalence of suicide attempts and injury by suicide attempt among Asian American female adolescents than among Asian American male adolescents from 1991 to 2019, although upon further analysis, the trends of these increases were not statistically significant. There was a significant increase in suicide plan among Asian American adolescents who were sexual minorities who had sex with same-sex partners or partners of both sexes. This study addresses the need to advance our understanding of sex and sexual orientation disparities in suicidal behaviors among Asian American adolescents. Screening for intersectional sex and sexual identity is crucial to identify the unique developmental needs of Asian American youth who are sexual minorities. Future research is warranted to examine race/ethnicity–specific and sexual orientation–specific risk and protective factors associated with youth suicide among Asian American populations and how social determinants can inform culturally relevant interventions. To prevent suicide among Asian American youth, culturally tailored efforts are needed, given that the one-size-fits-all solution may let inequality grow unchecked in our society. Continued policy efforts at the structural levels to reduce mental health stigma are warranted to increase the awareness of suicide risks among youth who are racial and sexual minorities.

## Notes

Supplement. **eTable 1.** Sample Demographic Characteristics **eTable 2.** Prevalence of Suicidal Behaviors by Sex (N = 7619) **eTable 3.** Logistic Regressions of Trends in Suicidal Behaviors by Sex, Sexual Identity, and Intersectionality **eFigure 1.** Annual Percentage Changes for Suicidal Behaviors **eFigure 2.** Annual Percentage Changes for Suicidal Behaviors by Intersections of Sex and Sexual Identity **eFigure 3.** Annual Percentage Changes for Suicidal Behaviors by Intersections of Sex and Sex of Sexual Contacts **eFigure 4.** Annual Percentage Changes for Suicidal Behaviors by Intersections of Sexual Identity and Sex of Sexual Contacts
