# Supplementary material for: Temporal Trends and Disparities in Suicidal Behaviors by Sex and Sexual Identity Among Asian American Adolescents
Source: JAMA Netw Open. 2021 Apr 16;4(4):e214498. doi: 10.1001/jamanetworkopen.2021.4498 (PMC8052595; doi:10.1001/jamanetworkopen.2021.4498)
Supplement: Supplement. — eTable 1. Sample Demographic Characteristics eTable 2. Prevalence of Suicidal Behaviors by Sex (N = 7619) eTable 3. Logistic Regressions of Trends in Suicidal Behaviors by Sex, Sexual Identity, and Intersectionality eFigure 1. Annual Percentage Changes for Suicidal Behaviors eFigure 2. Annual Percentage Changes for Suicidal Behaviors by Intersections of Sex and Sexual Identity eFigure 3. Annual Percentage Changes for Suicidal Behaviors by Intersections of Sex and Sex of Sexual Contacts eFigure 4. Annual Percentage Changes for Suicidal Behaviors by Intersections of Sexual Identity and Sex of Sexual Contacts [file jamanetwopen-e214498-s001.pdf]

Supplemental Online Content

Xiao Y, Lu W. Temporal trends and disparities in suicidal behaviors by sex and sexual identity among Asian American adolescents. *JAMA Netw Open*. 2021;4(4):e214498. doi:10.1001/jamanetworkopen.2021.4498

eTable 1. Sample Demographic Characteristics
eTable 2. Prevalence of Suicidal Behaviors by Sex (N = 7619)
eTable 3. Logistic Regressions of Trends in Suicidal Behaviors by Sex, Sexual Identity, and Intersectionality
eFigure 1. Annual Percentage Changes for Suicidal Behaviors
eFigure 2. Annual Percentage Changes for Suicidal Behaviors by Intersections of Sex and Sexual Identity
eFigure 3. Annual Percentage Changes for Suicidal Behaviors by Intersections of Sex and Sex of Sexual Contacts
eFigure 4. Annual Percentage Changes for Suicidal Behaviors by Intersections of Sexual Identity and Sex of Sexual Contacts

This supplemental material has been provided by the authors to give readers additional information about their work.

© 2021 Xiao Y et al. *JAMA Network Open.*

eTable 1. Sample Demographic Characteristics[a]

	Sample A (1991-2019) (n = 7619)		Sample B (2015-2019) (n = 1576)	
Sex, No. (%)				
Male	3859	52.9	766	50.8
Female	3760	47.1	810	49.2
Sexual Identity, No. (%)				
Heterosexual			1380	87.2
Sexual Minority			196	12.8
Sex of Sexual Contact, No. (%)				
Never/Opposite Sex			1516	96.8
Same/Both Sex			60	3.2
Grade Level, No. (%)				
9th	1814	25.0	412	26.3
10th	1801	25.5	358	23.6
11th	1943	24.8	427	24.6
12th	2061	24.7	379	25.5

[a] No. is unweighted. % are weighted, incorporated survey weights that account for probabilities of sample selection, nonresponse and oversampling.

© 2021 Xiao Y et al. *JAMA Network Open.*

eTable 2. Prevalence of Suicidal Behaviors by Sex (N = 7619)[a]

	Suicidal Ideation			Suicide Plan			Suicide Attempt			Injury by Suicide Attempt		
	Total	Female	Male	Total	Female	Male	Total	Female	Male	Total	Female	Male
**1991**	34.4	47.4	21.9	24.7	35.8	14.3	12.5	15.5	9.7	1.3	1.2	1.5
**1993**	24.9	29.3	21.6	18.9	19.4	18.6	9.3	14.7	5.3	1.5	1.9	1.2
**1995**	20.1	29.2	14.1	19.1	24.1	15.8	7.6	7.5	7.5	0.3	0.8	0.1
**1997**	21.1	25.6	17.5	16.0	19.1	13.7	6.9	11.4	3.3	1.5	1.3	1.6
**1999**	21.7	28.8	15.6	17.9	27.3	10.0	7.0	9.3	5.1	1.9	3.8	0.3
**2001**	18.5	22.5	14.5	18.1	24.8	11.4	10.0	13.0	7.0	2.4	2.9	1.8
**2003**	21.2	27.5	16.2	24.5	30.8	19.4	14.0	18.6	10.0	5.6	6.5	5.0
**2005**	15.9	20.4	11.9	13.3	15.7	11.3	6.9	9.4	4.9	1.9	3.0	1.1
**2007**	11.2	13.7	9.1	10.8	11.2	10.5	5.6	5.0	6.2	2.1	2.0	2.3
**2009**	14.9	16.9	12.9	12.6	14.7	10.4	4.0	3.7	4.3	1.4	0.7	2.0
**2011**	18.9	21.1	17.1	14.4	15.6	13.3	10.8	15.0	7.0	4.5	5.1	4.0
**2013**	16.7	22.9	10.2	15.8	17.9	13.5	9.5	11.7	7.1	3.7	3.3	4.1
**2015**	17.7	21.3	14.9	13.8	19.7	8.9	7.8	11.1	5.2	1.5	2.4	0.6
**2017**	17.4	17.8	17.0	16.1	18.3	13.7	5.7	8.4	2.7	2.7	4.0	1.2
**2019**	19.7	22.0	17.3	16.1	19.2	13.0	7.7	8.4	7.1	1.7	1.9	1.4
**Changes 1991-2019** [b]	-42.6%	-53.6%	-21.2%	-34.5%	-46.3%	-8.7%	-38.1%	-45.7%	-26.9%	24.1%	58.9%	-3.4%
**Changes 2009-2019** [b]	32.0%	29.8%	34.1%	28.2%	30.7%	24.9%	94.4%	126.8%	67.4%	22.4%	165.3%	-29.7%

[a] Weighted percentages.
[b] Percent changes.

© 2021 Xiao Y et al. *JAMA Network Open.*

eTable 3. Logistic Regressions of Trends in Suicidal Behaviors by Sex, Sexual Identity, and Intersectionality[a]

	Suicidal Ideation		Suicide Plan		Suicide Attempts		Injury by Suicide	
	Adjusted OR	95% CI	Adjusted OR	95% CI	Adjusted OR	95% CI	Adjusted OR	95% CI
**Sex**								
Female (Ref.=Male)	0.96**	0.93-0.99	0.98	0.94-1.02	0.98	0.93-1.04	0.99	0.89-1.09
**Sexual Identity**								
Sexual Minority (Ref.=Heterosexual)	1.42	0.96-2.09	0.81	0.55-1.21	1.10	0.54-2.25	0.54	0.15-1.96
**Sex of Sexual Contact**								
Same/Both Sex (Ref.=Never/Opposite Sex)	1.61	0.71-3.62	1.29	0.56-2.95	1.63	0.63-4.24	1.32	0.39-4.51
**Sex x Sexual Identity**								
Sexual Minority Male	1.29	0.61-2.74	0.94	0.38-2.29	1.49	0.44-5.07	0.16	0.02-1.15
Heterosexual Female	0.86	0.57-1.30	0.82	0.50-1.35	0.82	0.35-1.95	0.36	0.08-1.69
Sexual Minority Female	1.34	0.80-2.27	0.65	0.39-1.10	0.84	0.31-2.25	0.29	0.04-2.13
**Sex x Sex of Sexual Contact**								
Same/Both Sex Male	3.64*	1.23-10.72	2.61	0.77-8.87	1.41	0.21-9.58	NA	NA
Never/Opposite Sex Female	1.07	0.71-1.61	0.94	0.56-1.56	0.79	0.36-1.75	0.26	0.05-1.30
Same/Both Sex Female	1.18	0.38-3.65	0.97	0.33-2.91	1.75	0.49-6.23	0.71	0.11-4.58
**Sexual Identity x Sex of Sexual Contact**								
Same/Both Sex Heterosexual Male	3.28	0.92-11.78	1.98	0.51-7.75	2.71	0.66-11.05	2.17	0.50-9.35
Never/Opposite Sex Sexual Minority	1.74*	1.13-2.67	0.88	0.57-1.34	1.13	0.48-2.63	1.00	1.00-1.00
Same/Both Sex Sexual Minority	2.51	0.98-6.42	1.40	0.48-4.11	1.49	0.51-4.35	0.84	0.18-3.92

Note. OR, odds ratio; 95% CI, 95% confidence interval. Ref., reference. NA, not applicable. The significance threshold was P < .05 and the testing was 2-sided. All models controlled for the grade levels. All estimates shown were based on a series of models with interactions between each demographic characteristic and the survey year terms to determine whether trends of suicidal behaviors over time differed between demographic subgroups. All analyses incorporated the complex sampling design and survey weight to obtain the US nationally representative estimates, accounting for probabilities of sample selection, survey nonresponse, and oversampling of Black and Hispanic students.

© 2021 Xiao Y et al. *JAMA Network Open.*

eFigure 1. Annual Percentage Changes for Suicidal Behaviors

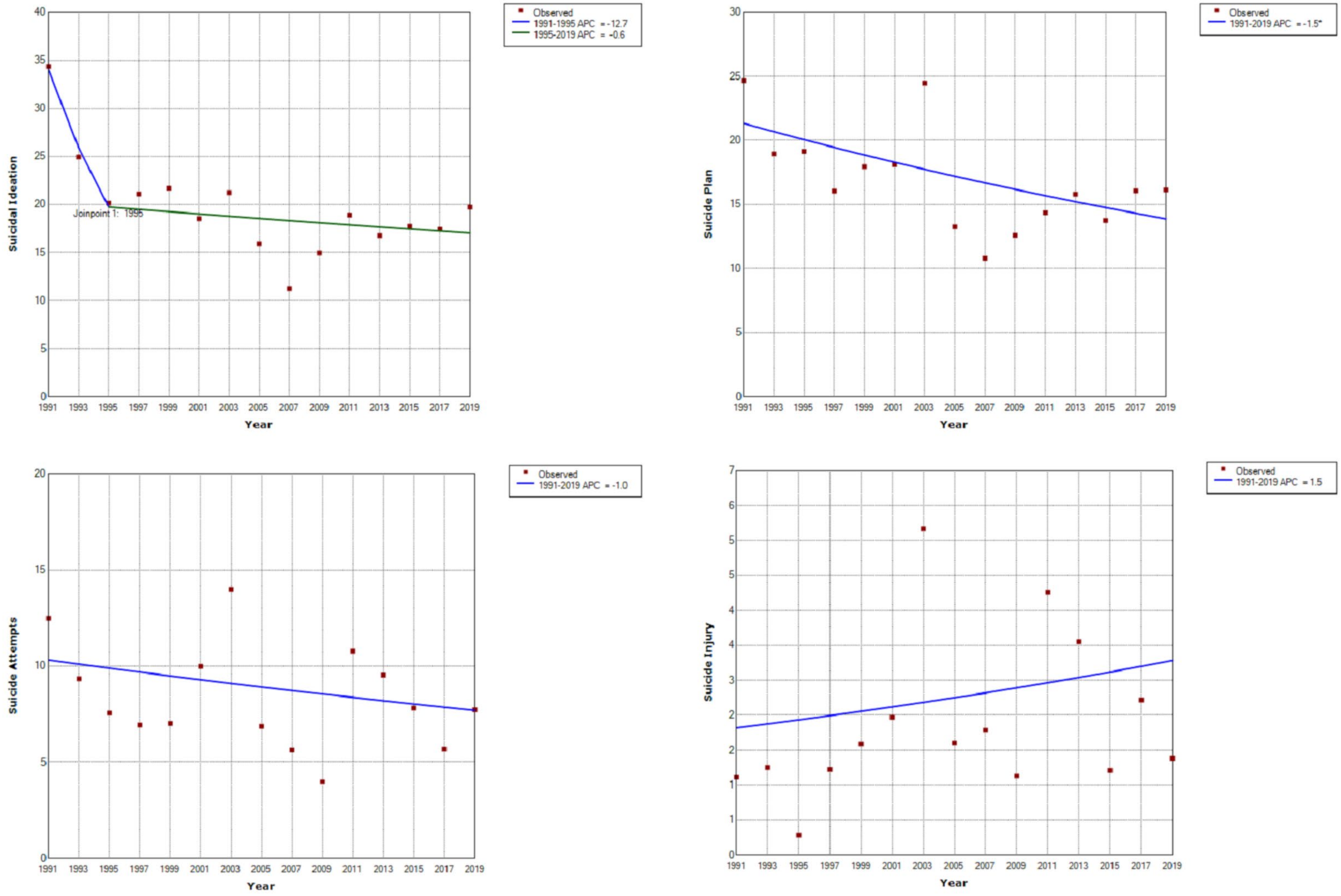

© 2021 Xiao Y et al. *JAMA Network Open.*

eFigure 2. Annual Percentage Changes for Suicidal Behaviors by Intersections of Sex and Sexual Identity

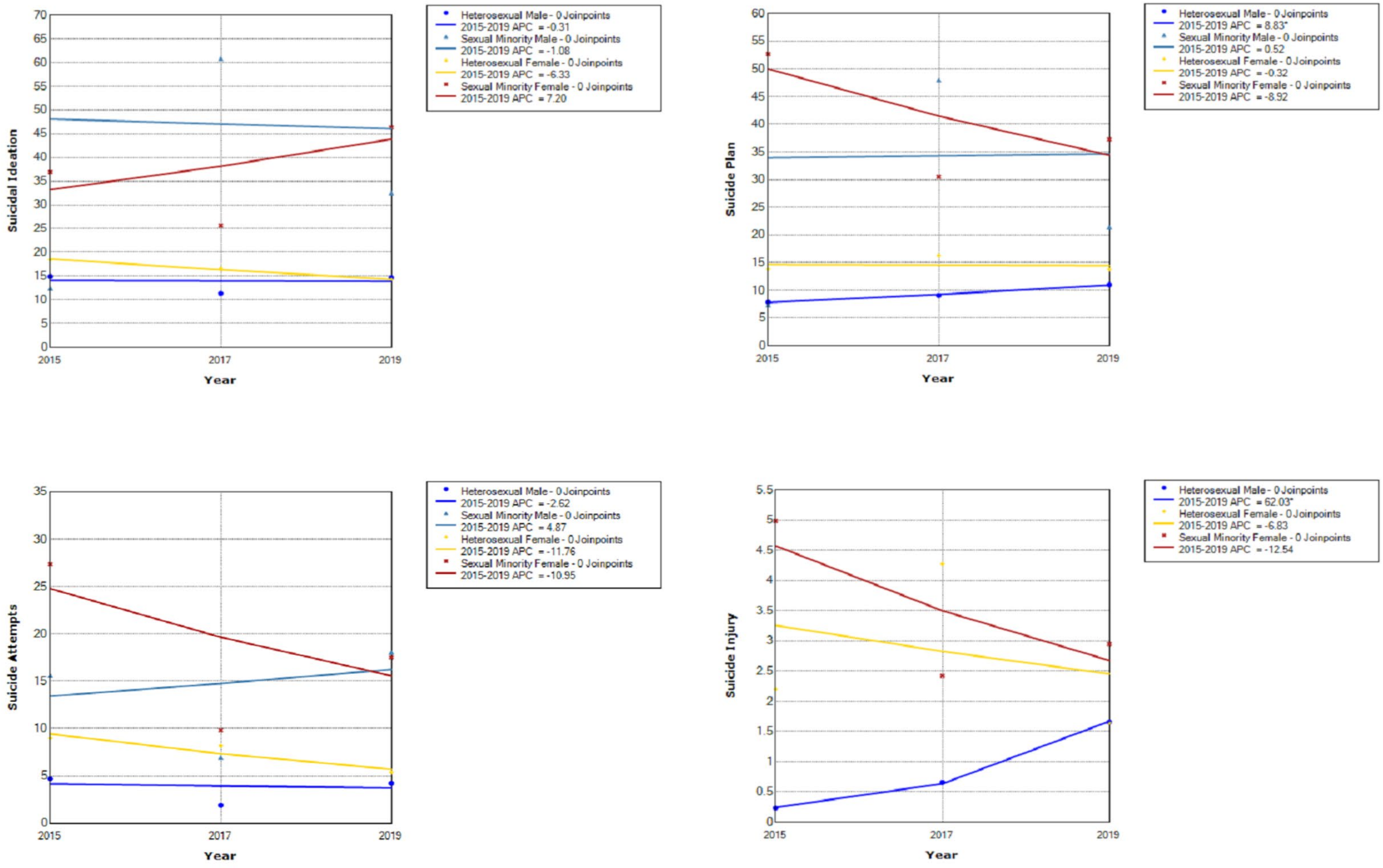

© 2021 Xiao Y et al. *JAMA Network Open.*

eFigure 3. Annual Percentage Changes for Suicidal Behaviors by Intersections of Sex and Sex of Sexual Contacts

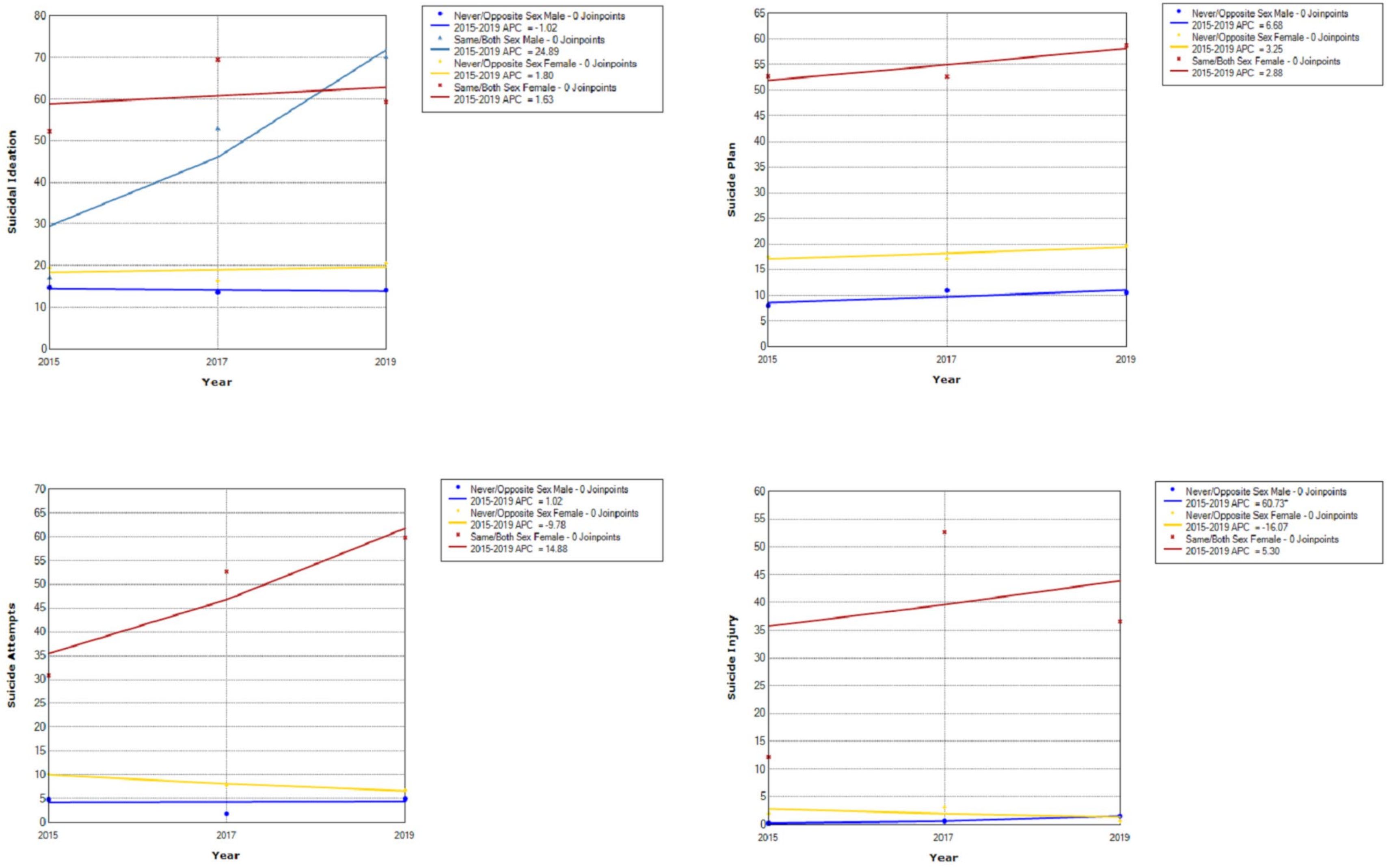

© 2021 Xiao Y et al. *JAMA Network Open.*

eFigure 4. Annual Percentage Changes for Suicidal Behaviors by Intersections of Sexual Identity and Sex of Sexual Contacts

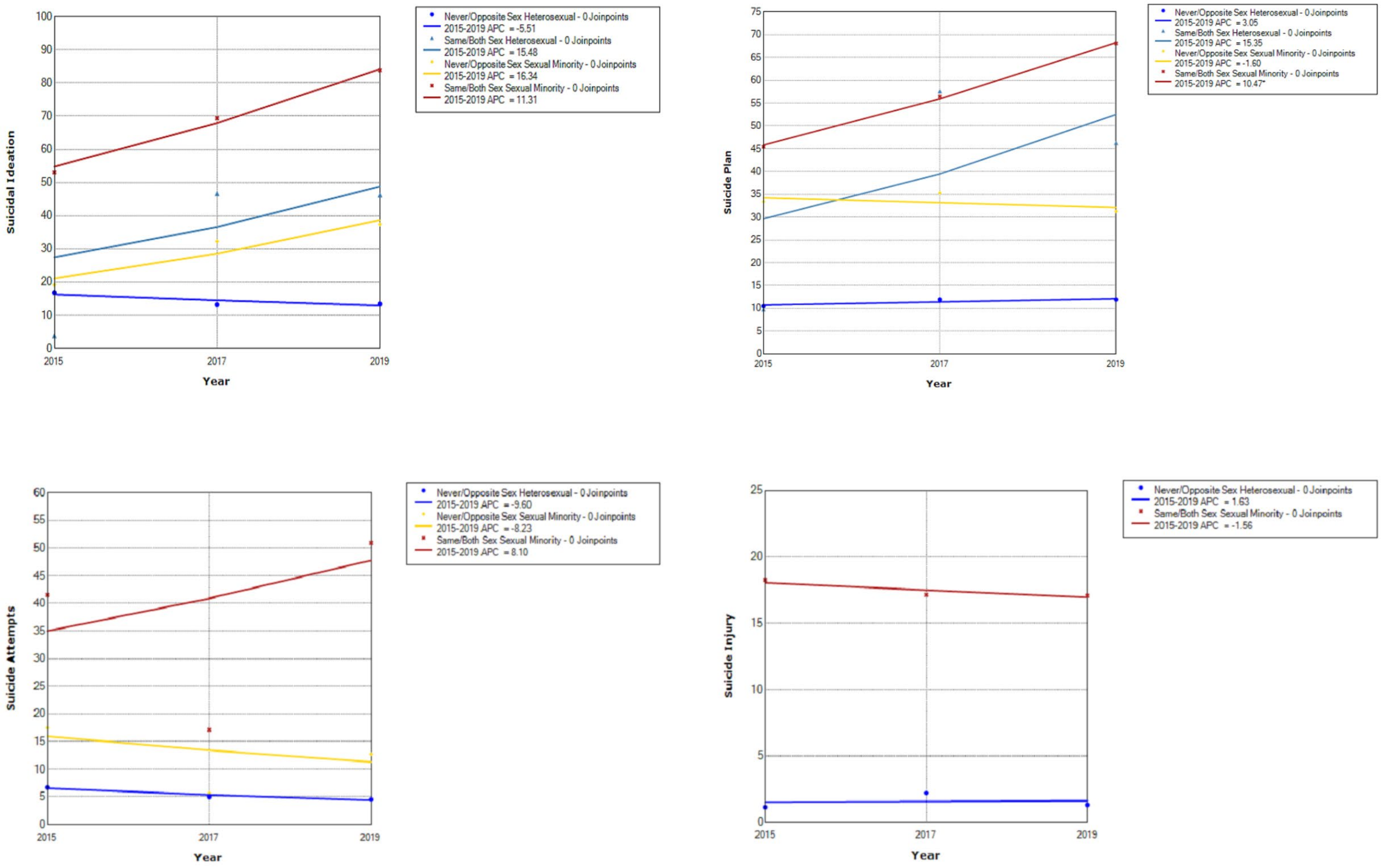

© 2021 Xiao Y et al. *JAMA Network Open.*
